# Toxicity and Long-Term Outcomes of Dose-Escalated Intensity Modulated Radiation Therapy to 74Gy for Localised Prostate Cancer in a Single Australian Centre

**DOI:** 10.3390/cancers3033419

**Published:** 2011-09-01

**Authors:** Joseph Sia, Daryl Lim Joon, Angela Viotto, Carmel Mantle, George Quong, Aldo Rolfo, Morikatsu Wada, Nigel Anderson, Maureen Rolfo, Vincent Khoo

**Affiliations:** 1 Austin Health Radiation Oncology Centre, Heidelberg Repatriation Hospital, 300 Waterdale Road, Heidelberg West, Victoria 3081, Australia; E-Mails: joseph.sia@tablecolorworks.com (J.S.); daryl.limjoon@austin.org.au (D.L.J.); angela.viotto@austin.org.au (A.V.); carmel.mantle@austin.org.au (C.M.); gquong@radoncvic.com.au (G.Q.); arolfo@radoncvic.com.au (A.R.); morikatsu.wada@austin.org.au (M.W.); nigel.anderson@austin.org.au (N.A.); maureen.rolfo@austin.org.au (M.R.); 2 Radiation Oncology Victoria, East Melbourne, Victoria 3002, Australia; 3 Department of Medicine, University of Melbourne, Melbourne Victoria 3053, Australia; 4 Royal Marsden Hospital & Institute of Cancer Research, London SW3 6JJ, UK

**Keywords:** dose escalation, IMRT, late effects, prostate cancer, toxicity, radiation

## Abstract

**Purpose:**

To report the toxicity and long-term outcomes of dose-escalated intensity-modulated radiation therapy (IMRT) for patients with localised prostate cancer.

**Methods and Materials:**

From 2001 to 2005, a total of 125 patients with histologically confirmed T1-3N0M0 prostate cancer were treated with IMRT to 74Gy at the Austin Health Radiation Oncology Centre. The median follow-up was 5.5 years (range 0.5–8.9 years). Biochemical prostate specific antigen (bPSA) failure was defined according to the Phoenix consensus definition (absolute nadir + 2ng/mL). Toxicity was scored according to the RTOG/EORTC criteria. Kaplan-Meier analysis was used to calculate toxicity rates, as well as the risks of bPSA failure, distant metastases, disease-specific and overall survival, at 5 and 8-years post treatment.

**Results:**

All patients completed radiotherapy without any treatment breaks. The 8-year risks of ≥ Grade 2 genitourinary (GU) and gastrointestinal (GI) toxicity were 6.4% and 5.8% respectively, and the 8-year risks of ≥ Grade 3 GU and GI toxicity were both < 0.05%. The 5 and 8-year freedom from bPSA failure were 76% and 58% respectively. Disease-specific survival at 5 and 8 years were 95% and 91%, respectively, and overall survival at 5 and 8 years were 90% and 71%, respectively.

**Conclusions:**

These results confirm existing international data regarding the safety and efficacy of dose-escalated intensity-modulated radiation therapy for localised prostate cancer within an Australian setting.

## 1. Introduction

There has been significant attention over the past decade on dose escalation of radiotherapy for localised prostate cancer on the premise of a dose response relationship. The opportunity to shape treatment beams in three dimensions (3D) for conformal radiotherapy (3DCRT) has been shown to significantly reduce radiotherapy related side-effects in a randomised study [1]. In turn, 3DCRT has permitted the initiation of several randomised studies of dose escalation for prostate cancer [2-5]. These randomised trials demonstrate that the delivery of higher doses (74–78 Gy) than conventionally prescribed (64–68 Gy) has been associated with improved biochemical prostate specific antigen (bPSA) control rates. Some studies have also reported an increase in freedom from distant metastasis [6,7]. A recent meta-analysis of randomised controlled trials evaluating dose escalation for prostate radiotherapy confirmed a positive relationship between radiation dose and bPSA control across all risk categories of prostate cancer [8]. This meta-analysis estimated a reduction in bPSA failure risk of approximately 1.8% for each 1 Gy increase in dose.

However, despite 3DCRT and optimisation of its techniques [9-11], the increase in dose has also resulted in an increase in rectal toxicity [3-5,8,12]. Intensity-modulated radiotherapy (IMRT) has been shown to be one approach in overcoming this. IMRT is a technological refinement of 3DCRT that delivers non-uniform beam intensities and therefore can sculpt the high dose distributions around complex target volumes, particularly concave volumes where the organ-at-risk (OAR) such as the rectum is located within the target concavities and thus overcome one of the limitations of 3DCRT. Prostate radiotherapy studies utilising IMRT have reported that this technique can achieve better rectal conformality and lower rectal toxicity, even with higher doses beyond 78 Gy [13-20].

Dose-escalation with 3DCRT or IMRT has since become the standard treatment in many radiotherapy centres for prostate radiotherapy. To date there remains a dearth of data on outcomes in the Australasian scenario of dose-escalated radiotherapy for localised prostate cancer. The Austin Health Radiation Oncology Centre in Victoria was one of the early adopters of both dose-escalation and IMRT in treating localised prostate cancer in Australia. Dose-escalation to 74 Gy with IMRT was introduced in the department in 2001, and was subsequently superseded by escalation to 78 Gy in 2006. In this study, we have evaluated our cohort of 125 patients treated prospectively to 74 Gy with IMRT in this department and report on the toxicity, failure and survival outcomes.

## 2. Materials and Methods

### 2.1. Patient Characteristics

Between 2001 and 2005, 125 patients with biopsy-proven, localised adenocarcinoma of the prostate (T1-3N0M0) were treated with definitive IMRT to 74Gy at the Austin Health Radiation Oncology Centre. Staging was completed using magnetic resonance imaging (MRI) of the pelvis and bone scan and was based on the 1998 American Joint Commission on Cancer (AJCC) staging system [21]. Risk stratification was according to the National Comprehensive Cancer Network (NCCN) classification of risk groups [22]. The median age was 69 years, with a range of 47–80 years. Disease characteristics of the patient cohort are outlined in Table 1. Nearly two-thirds (65%) of the cohort comprised patients in the high to very-high risk NCCN risk category.

### 2.2. Treatment

All patients were seen and treated by the same radiation oncologist in the department. Androgen deprivation therapy (ADT) was used in accordance with the clinical protocol of the treating clinician. This was guided by the risk category, the presence of other high risk features, and the presence of co-morbidities. Patients in the intermediate and high risk groups received 3 months of neoadjuvant ADT. If ADT was well tolerated, intermediate risk patients would continue with concurrent and adjuvant ADT for a total of 3 months. High risk patients would continue with concurrent and adjuvant ADT for a total of 3 years.

During simulation and treatment, patients were treated supine and immobilized with a MedTec HipFix board and a custom-made two-part foam extending from the iliac crest to mid-thigh. The head was supported with a double square sponge and ankles were supported with a SimMed FeetFix device. To minimize variations in bladder and bowel volumes, all patients were instructed to take a Microlax enema either the night before or the morning of the simulation appointment, as well as for the first ten treatment days, and to drink 2 cups of water holding approximately 250–300 mL just before all radiotherapy appointments. In addition, the patients were instructed to take Benefibre daily throughout the treatment period. Patients were exempted from Benefibre or Microlax enemas if they reported problematic loose bowel motions or diarrhoea.

All patients underwent whole pelvic MRI for co-registration with the simulation computed tomography (CT) unless they had contraindications. Acquisition of MRI was performed in the treatment position. Treatment is delivered with a single phase 5–7 field IMRT technique incorporating a simultaneous integrated boost. The clinical target volume (CTV) to receive a mean dose of 54Gy (CTV-1) was defined as the hybrid of CT and MRI seminal vesicle glands and prostate gland contours. The CTV to receive a mean dose of 74 Gy (CTV-2) was defined as the hybrid of CT and MRI prostate gland only contours. A margin of 1cm in all directions except posteriorly, where the margin was 0.6 cm, was added around the CTV's to create the respective planning target volumes (PTV). The rectal volume was defined as the rectum plus its contents, contoured from 1.0 cm above to 1.0 cm below the PTV-2.

**Figure 1.**
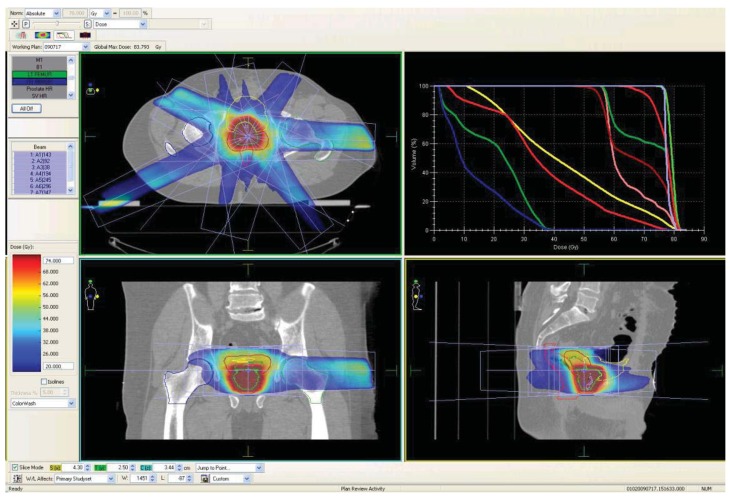
Intensity modulated radiation therapy (IMRT) planning for a typical prostate case.

The dose constraints were specified such that no more than 60% of the rectum received ≥40 Gy, no more than 50% of the bladder received ≥50 Gy, and no more than 50% of the femora received ≥50 Gy. Maximum point doses allowed on the small and large bowel were 50 Gy and 60 Gy, respectively. 99% of the PTV-1 must receive ≥51.30 Gy, and 95% of the PTV-2 must receive ≥70.30 Gy. Figure 1 illustrates a typical IMRT prostate case planned in the department.

### 2.3. Follow-up and Analysis of Endpoints

The median follow-up was 5.5 years (66 months), with a range of 0.5 to 8.9 years. Patients were reviewed weekly during treatment, at two weeks following completion of radiotherapy, 3–4 monthly in years 1–2, 6 monthly in years 3–4, then yearly thereafter. Bladder and bowel toxicity scores were prospectively scored by clinicians according to the Radiation Therapy Oncology Group and the European Organization for Research and Treatment of Cancer (RTOG/EORTC) criteria [23]. Toxicity developing from day 1 of commencement of radiotherapy to day 90 was considered acute and late toxicity was measured from day 91 onwards. Biochemical PSA failure was defined as a 2 ng/mL rise from the PSA nadir, in accordance to the Phoenix consensus definition [24].

The endpoints of this report are ≥ Grade 2 acute and late genitourinary (GU) and gastrointestinal (GI) toxicity, freedom from biochemical PSA failure (bFFF), freedom from distant metastases, overall survival, and disease-specific survival. Due to limited patient numbers in the low and very-high risk groups, the low and intermediate risk groups, as well as the high and very-high risk groups, were respectively combined for risk group analysis. Actuarial risks were calculated by the Kaplan-Meier method and are reported at 5 and 8 years post-treatment.

## 3. Results

### 3.1. Acute and Late Toxicity

**Figure 2.**
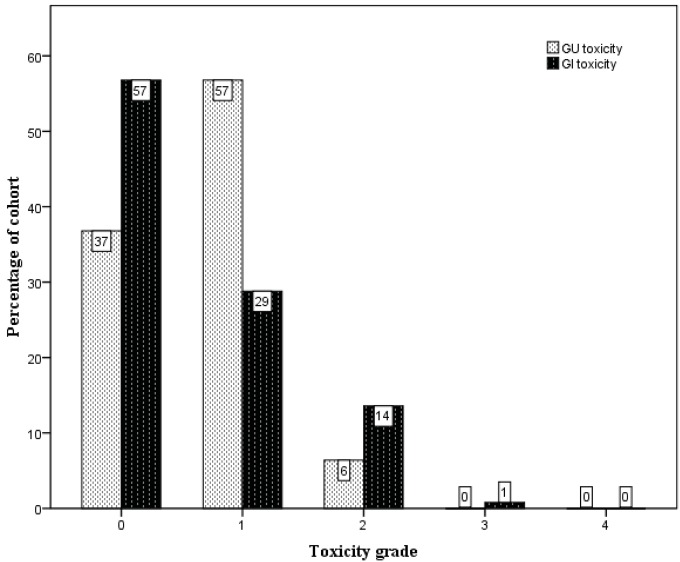
Crude incidences of acute genitourinary (GU) and gastrointestinal (GI) toxicity.

The crude numbers of the incidences of acute genitourinary (GU) and gastrointestinal (GI) toxicity are presented in Figure 2. Specifically, the incidences of ≥ Grade 2 acute GU and GI toxicity were 6% and 14%, respectively. One patient was scored to have Grade 3 acute GI toxicity when he developed severe diarrhoea and pain on defecation immediately following completion of treatment, requiring admission for management. He was noted to have a background history of ulcerative colitis, and sigmoidoscopy confirmed changes consistent with proctitis. His symptoms settled well with time and there was no long-term sequela from this. There was no acute Grade 3 GU toxicity.

**Figure 3.**
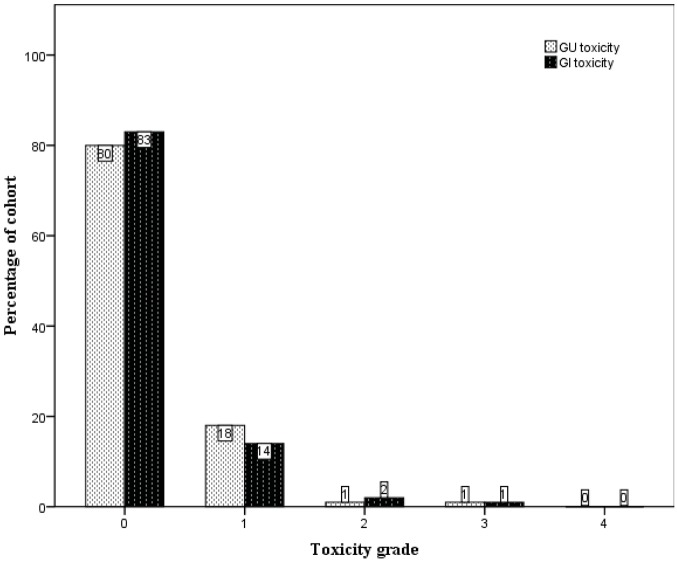
Crude incidences of late genitourinary (GU) and gastrointestinal (GI) toxicity.

Late treatment-related toxicity was uncommon. The crude numbers of their incidences are presented in Figure 3. The large majority of patients did not experience any late GU or GI toxicity (80% and 83% respectively). One patient developed gross haematuria requiring blood transfusions five years subsequent to completion of radiation therapy, with cystoscopy showing changes consistent with radiation-induced cystitis. However, in the following year it became evident that the patient had a superficial urothelial cell carcinoma arising from the right kidney pelvis. He was scored to have Grade 3 late GU toxicity. Another patient was scored to have Grade 3 late GI toxicity when he developed persistent rectal bleeding after radiotherapy. He was noted to be on warfarin for a previous stroke and pulmonary embolism. On consultation with the colorectal surgeons, he was kept on warfarin despite the rectal bleeding, but underwent argon laser therapy, which provided good effect.

**Figure 4.**
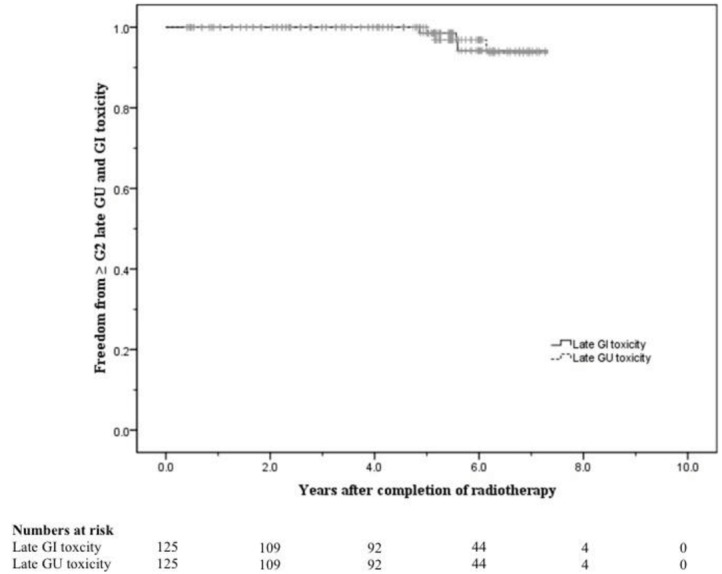
Freedom from ≥ Grade 2 late genitourinary (GU) and gastrointestinal (GI) toxicity.

The 5-year actuarial risks of developing ≥ Grade 2 late GU and GI toxicity were 2.2% and 1.4% respectively. At 8 years these were 6.4% and 5.8% respectively (Figure 4).

### 3.2. Freedom from Failure and Survival

**Figure 5.**
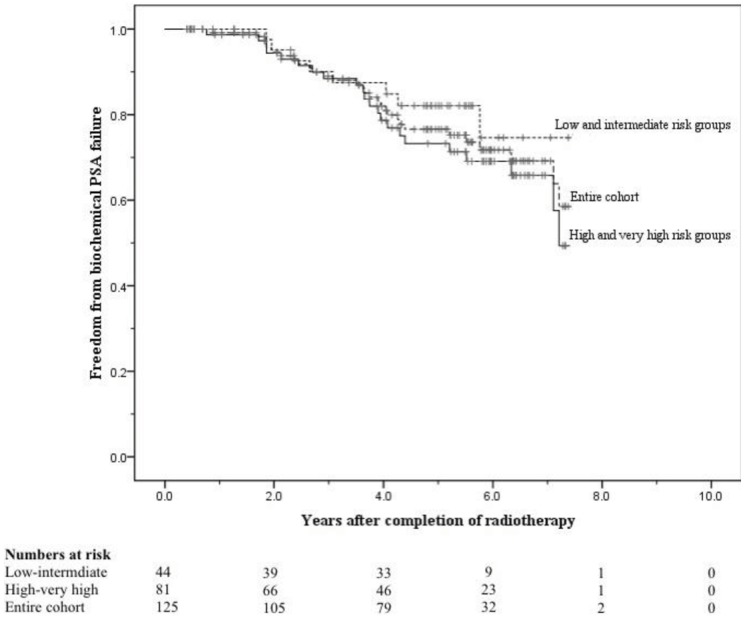
Freedom from biochemical PSA failure for the low and intermediate risk groups, high and very high risk groups, and the entire cohort according to the Phoenix consensus definition

**Figure 6.**
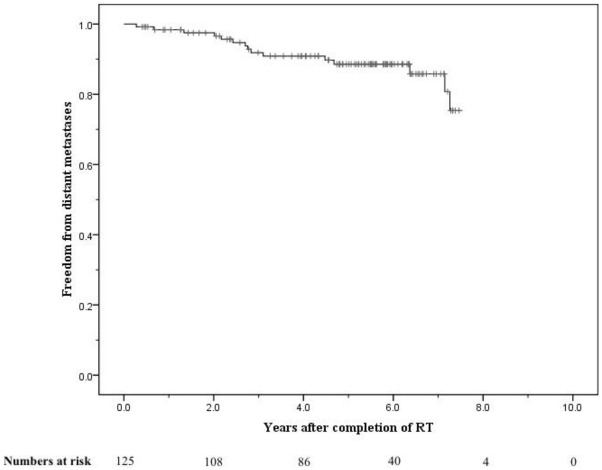
Freedom from distant metastases for the entire cohort.

The crude numbers of patients with biochemical, local, nodal and distant failure were 30, 1, 5, and 13 respectively. At 5 years the post-treatment freedom from biochemical failure (bFFF) and freedom from distant metastases were 76% and 89% respectively (Figures 5 and 6).

**Figure 7.**
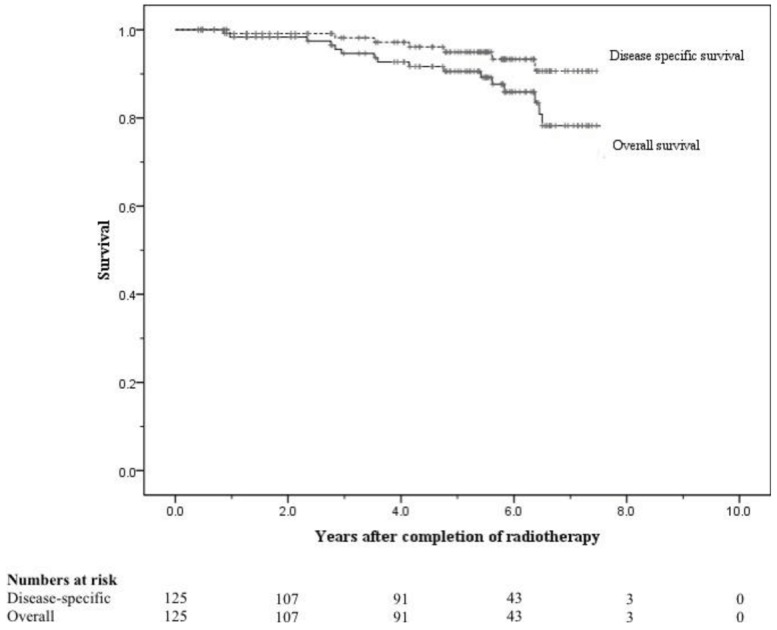
Disease-specific survival and overall survival for the entire cohort.

At 8 years these were 58% and 75% respectively (Figures 5 and 6). When analysed by risk group, at 5 years bFFF was 82% for the low and intermediate risk groups, and 73% for the high and very-high risk groups (Figure 5). At 8 years these were 75% and 49% respectively (Figure 5). The 5-year disease-specific survival and overall survival were 95% and 90% respectively (Figure 7). At 8 years the disease-specific survival and overall survival were 91% and 71% respectively (Figure 7).

## 4. Discussion

The long term results of our single centre study corroborate the safety and efficacy of dose escalation using IMRT for localised prostate cancer. The toxicity experienced by the men treated at our centre with radical radiotherapy supports the growing evidence for the benefit of IMRT in rectal toxicity avoidance. The prospective non-randomised study from Memorial Sloan Kettering Cancer Center (MSKCC) treating men with prostate cancer to 81 Gy using IMRT reported 8-year risks of ≥ Grade 2 GU and GI risks of 15% and 1.6%, respectively [20]. These toxicity grades were scored according to the Common Terminology Criteria for Adverse Events Version 3.0 (CTCAE). When the radiation dose was escalated to an ultra-high dose of 86.4 Gy, the same centre reported 5-year risks of ≥ Grade 2 GU and GI toxicity of 16% and 3% respectively [14]. This compares to 5-10 year risks with dose-escalated 3DCRT of 11-39% for GU toxicity and 23-38% for GI toxicity, based on RTOG criteria [2-5]. A dose-escalation study to 74 Gy with 3DCRT in New South Wales, Australia, has reported early 3-year risks of 17.3% and 9.2% for ≥ Grade 2 GU and GI toxicity, respectively [25].

Our rates of acute and late GU toxicity are much lower than those reported by the aforementioned groups. As our toxicity data is prospectively collected, this could not have accounted for the difference. In addition, we had strict adherence to a conservative set of normal tissue constraints using the organ-at-risk (OAR) wall rather than solid organ parameters and a policy of compromise of target coverage to maintain these OAR constraints. Another reason could be the limitation of the RTOG/EORTC scale, which does not include evaluation of ‘bothersome’ symptoms such as urgency and incontinence. It has been reported that the RTOG/EORTC scale, when compared to the CTCAE scale, can result in an underreporting of GU toxicity of up to 10% [26] and for GI toxicity, other toxicity assessment systems have been suggested to be more reliable [27,28].

It should be borne in mind that our patient cohort has a prostate disease distribution of mainly high to very-high risk categories. Furthermore, at 8 years post-treatment the remaining number of cases at risk was limited. Therefore at this stage a small number of events would result in large changes in the actuarial risks. The reported 5-year risks would arguably be considered more representative.

With these factors considered, the failure and survival rates of our study are comparable with those data reported internationally. The MSKCC experience with IMRT to 81Gy did not report an overall bFFF, but their 8-year bFFF were 89%, 78% and 67% for low, intermediate and high-risk groups respectively [20]. The M.D. Anderson dose-escalation series on the other hand reported overall 8-year bFFF of 59% and 78% for 70 Gy and 78 Gy, respectively, using an initial four-field box technique to 46 Gy [2]. The Netherlands dose-escalation trial to 78 Gy with 3DCRT reported a 7-year bFFF of 56% [4], and the MRC RT01 trial reported a 5-year bFFF of 71% with 74 Gy 3DCRT [5].

There has been the question of cost-effectiveness for IMRT compared to 3DCRT in the treatment of prostate cancer. IMRT is commonly believed to be labour-intensive and therefore incurs an added cost arising from additional medical, radiographer and physics staff time, but is nonetheless superior to 3DCRT in avoidance of organs at risk [17,29]. Whilst these additional resources are needed with IMRT and its quality assurance, there is a longer term benefit to survivors of prostate cancer with their improved quality of life from curative therapy and the avoidance of treatment for late radiotherapy-related side-effects. At present there are no randomised control trials comparing the two techniques, and the Australian & New Zealand Faculty of Radiation Oncology Genito-Urinary Group 2010 consensus guidelines have not recommended one over the other [30]. A recent evaluation by Hummel *et al.* for the National Institute for Health Research (NIHR) in UK represents one of the first major attempts at costing IMRT for prostate cancer. While the report emphasised on the many uncertainties in establishing the cost-effectiveness of IMRT when compared to 3DCRT, it nonetheless concluded that IMRT is likely to be cost-effective especially when a reduction in late GI toxicity of 15% is assumed, taking into account factors such as the familiarity of the department with IMRT [31].

The single radiation oncologist treating the entire cohort in our centre provides consistency and improves our study's internal validity. All our patients also underwent MRI fusion with CT imaging during treatment planning. This has been shown to improve the accuracy of prostate and seminal vesicle delineation in the treatment of prostate cancer, and may also allow better sparing of erectile tissues [32-35]. This study however was not designed to investigate this. The main limitations of our study are the moderately small sample size, as well as the duration and consistency of follow-up, resulting in limited numbers nearing the 8-year mark. The main reasons for our patients being lost to follow-up were a change in the public health network, the moving of patient residences interstate, and the transfer to the care of private practice.

## 5. Conclusions

This study is to our knowledge the first major description of long-term outcome and toxicity of dose-escalated IMRT for localised prostate cancer in Australia. Our results using IMRT to 74 Gy are consistent with international data reporting good disease control, even with our high risk prostate cancer groups with minimal long-term toxicity. Our step-wise initiation of IMRT with careful quality assurance in a small Australian centre demonstrates the ability to incorporate high end technology in routine clinical practice and has permitted further IMRT dose-escalation to 78 Gy, which we hope to report in due course.

## Figures and Tables

**Table 1.** Patient Characteristics.

	**N**	**%**
T-stage		
T1	25	(20)
T2	57	(45)
T3	37	(30)
T4	6	(5)
Gleason score		
2–6	40	(32)
7	60	(48)
8–10	25	(20)
Pre-treatment PSA (ng/mL)		
<10	35	(28)
10–20	42	(34)
>20	48	(38)
Risk group		
Low	5	(4)
Intermediate	39	(31)
High	74	(59)
Very high	7	(6)

PSA = prostate-specific antigen
